# The impact of the COVID-19 pandemic on community prescription of opioid and antineuropathic analgesics for cancer patients in Wales, UK

**DOI:** 10.1007/s00520-023-07944-8

**Published:** 2023-08-22

**Authors:** Jun Han, Martin Rolles, Fatemeh Torabi, Rowena Griffiths, Stuart Bedston, Ashley Akbari, Bruce Burnett, Jane Lyons, Giles Greene, Rebecca Thomas, Tamsin Long, Cathy Arnold, Dyfed Wyn Huws, Mark Lawler, Ronan A Lyons

**Affiliations:** 1 Population Data Science, Swansea University Medical School, Swansea, UK; 2 DATA-CAN, the UK’s Health Data Research Hub for Cancer, London, UK; 3 South West Wales Cancer Centre, Swansea Bay University Health Board, Swansea, UK; 4 Welsh Cancer Intelligence and Surveillance Unit, Public Health Wales, Cardiff, UK; 5 Observatory and Cancer Analysis Team, Public Health Wales, Cardiff, UK; 6 Data Services, University of Leeds, Leeds, UK; 7 Patrick G Johnston Centre for Cancer Research, Queens University Belfast, Belfast, UK

**Keywords:** COVID-19 pandemic, Cancer, Pain, Prescription, Primary care, Analgesia

## Abstract

**Purpose:**

Public health measures instituted at the onset of the COVID-19 pandemic in the UK in 2020 had profound effects on the cancer patient pathway. We hypothesise that this may have affected analgesic prescriptions for cancer patients in primary care.

**Methods:**

A whole-nation retrospective, observational study of opioid and antineuropathic analgesics prescribed in primary care for two cohorts of cancer patients in Wales, using linked anonymised data to evaluate the impact of the pandemic and variation between different demographic backgrounds.

**Results:**

We found a significant increase in strong opioid prescriptions during the pandemic for patients within their first 12 months of diagnosis with a common cancer (incidence rate ratio (IRR) 1.15, 95% CI: 1.12–1.18, *p* < 0.001 for strong opioids) and significant increases in strong opioid and antineuropathic prescriptions for patients in the last 3 months prior to a cancer-related death (IRR = 1.06, 95% CI: 1.04–1.07, *p* < 0.001 for strong opioids; IRR = 1.11, 95% CI: 1.08–1.14, *p* < 0.001 for antineuropathics). A spike in opioid prescriptions for patients diagnosed in Q2 2020 and those who died in Q2 2020 was observed and interpreted as stockpiling. More analgesics were prescribed in more deprived quintiles. This differential was less pronounced in patients towards the end of life, which we attribute to closer professional supervision.

**Conclusions:**

We demonstrate significant changes to community analgesic prescriptions for cancer patients related to the UK pandemic and illustrate prescription patterns linked to patients’ demographic background.

**Supplementary Information:**

The online version contains supplementary material available at 10.1007/s00520-023-07944-8.

## Introduction

Pain is common in cancer patients. Cancer, its treatment, and non-cancer co-morbidity can all cause pain [1, 2]. Pharmacological cancer pain management in the UK commonly emulates the WHO Analgesic Ladder [3], which advocates a proportionate, step-wise approach from non-opioid analgesics to weak opioids, followed by strong opioids for analgesia. Antineuropathic agents, including antidepressants and anticonvulsants, and other adjuvants, are also used for controlling pain in cancer patients [4–8].

With the emergence of the COVID-19 global pandemic (the pandemic), healthcare delivery and utilisation changed dramatically [9]. Public and professional uncertainties and anxieties, both in anticipation and during the height of the pandemic, affected how palliation was managed in the community [10]. In the UK, national guidelines included measures to minimise virus transmission to both patients and healthcare workers, incorporating reduced face-to-face consultations in primary and secondary care [11]. NHS England and NHS Wales issued guidance listing repeat prescriptions as one of the solutions to minimise patient exposure [12, 13]. There were also preparations for increased management of end-of-life care in the community [14], in anticipation of loss of hospital capacity.

We hypothesised that the onset of the pandemic impacted cancer patients’ analgesic prescriptions in community settings, based on adapted systematic healthcare responses, changes to health-seeking attitude and behaviour, incidence rates, and the accessibility of cancer care [15–23].

Population and district-scale studies have reported on the impact of the pandemic on opioid and other analgesic prescriptions [24, 25], but not specifically for cancer patients. We present a retrospective, observational study examining the impact of the pandemic on opioid and antineuropathic prescriptions in the Welsh primary care setting for both newly diagnosed cancer patients and patients who died from cancer. Using linked data, we also examined the association of patients’ socioeconomic status with their analgesic prescriptions before and during the pandemic.

## Methods

### Data extraction and inclusion criteria

We assessed the impact of the pandemic on analgesic prescription patterns focussing on opioids and antineuropathics (hereafter referred to as ‘analgesics’) for cancer patients in Wales, UK, population 3.2M, served by an autonomous Health Service providing free prescriptions. Anonymised individual-level, population-scale linkable data sources were used within the Secure Anonymised Information Linkage (SAIL) Databank, a Secure Data Environment containing national-level data on the population of Wales [26, 27].

**Fig. 1:**
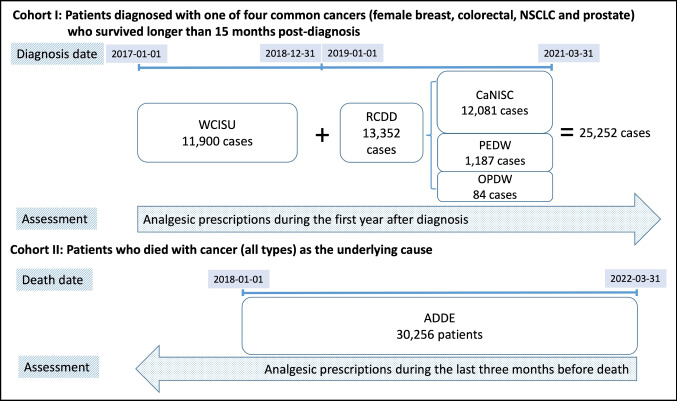
Two patient cohorts and the analgesic prescription assessment periods for each cohort. *WCISU*, Welsh Cancer Intelligence & Surveillance Unit data; *RCDD*, Rapid Cancer Diagnosis Dataset (Wales); *CaNISC*, Cancer Network Information System Cymru; *PEDW*, Patients Episodes Dataset for Wales; *OPDW*, Outpatient Database for Wales; *ADDE*, Annual District Death Extract

Two cohorts of cancer patients were included**.** Cohort I included patients diagnosed between 2017-01-01 and 2021-03-31 with one of four common cancers (female breast, colorectal, non-small cell lung (NSCLC), or prostate) who survived longer than 15 months post-diagnosis; the cut-off diagnosis date of 2021-03-31 was chosen to ensure that one-year prescription data after diagnosis was complete at the time of data extraction. Cohort II included patients who died with a malignant neoplasm as the underlying cause between 2018-01-01 and 2022-03-31 (Fig. 1).

Analgesic prescription data were extracted for the 12 months from the cancer diagnosis date for Cohort I patients and for the 3 months before the death date for Cohort II patients from the Welsh Longitudinal General Practice (WLGP) data (Fig. 1). WLGP contains prescribing and other GP event data from approximately 80% of all Wales general practices. The prescribing data included the exact date of prescription for each drug item and are coded using Read codes. Read codes for analgesics were classified into analgesic groups according to the UK Biobank ‘Primary care codings’ annotation in the May 2020 edition [28]. Medicine names and analgesic groups are shown in Supplementary Table 1.

Cohort I was selected to provide a relatively stable group of cancer patients (in terms of prescribing), in contrast to the end-of-Life patients in Cohort II. A 15-month survival period was used for Cohort I so that at least 12 months of prescribing data were available. This did not include the 3-month period defined as end-of-life care, which tends to have a different approach to analgesic prescriptions. Patients who experienced end-of-life care during the study are represented by Cohort II and data relating to the 3 months prior to their death (Fig. 1).

Diagnosis data for Cohort I patients, including cancer type and diagnosed stage, came from two sources (Cohort I in Fig. 1). Data for patients diagnosed between 2017-01-01 and 2018-12-31 came from the Welsh Cancer Intelligence & Surveillance Unit (WCISU) database, the National Cancer Registry for Wales. As a provider of official statistics, WCISU data are subject to approximately two years delay before release. To meet the urgent requirement of assessing the impact of the pandemic on cancer care and services in Wales, the Rapid Cancer Diagnosis Dataset (RCDD) was created, providing data for patients diagnosed from 2019-01-01 onwards. In RCDD, records of newly diagnosed patients in secondary care inpatient hospital admissions data (Patients Episodes Dataset for Wales, PEDW) and outpatient appointments data (Outpatients Database for Wales, OPDW) were added to the list of newly diagnosed cases recorded in the Welsh national electronic clinical cancer patient record system (Cancer Network Information System Cymru, CaNISC), to achieve data completeness for newly diagnosed cases. The following International Classification of Diseases version 10 (ICD-10) codes were used for cancer type extraction: female breast cancer (C50), colorectal cancer (C18, C19, C20), NSCLC (C33, C34), and prostate cancer (C60).

For Cohort II, records of all patients who died with a cancer (ICD-10 codes C00-C97 excluding C44) as the underlying cause were extracted from the Annual District Death Extract (ADDE), which contains the information of all deaths relating to Welsh residents.

Demographic information included week of birth, sex, and deprivation based on place of residence as assessed by the Lower-layer Super Output Area (LSOA) version 2011, linked to the socioeconomic status for each individual's Welsh Index of Multiple Deprivation (WIMD) version 2019 (Welsh Demographic Service Dataset, WDSD) with health factor of the local areas excluded [29]. Only patients with both a Welsh residence address and a Welsh GP registration at diagnosis (for Cohort I) and patients who died as Welsh residents (for Cohort II) were included. Only adult patients were included in both cohorts: aged 18 or older at diagnosis for Cohort I and aged 18 or older at death for Cohort II.

Patients’ comorbidities were extracted and counted from disease and symptom records contained in the WLGP data using the Elixhauser categorisation [30]. For Cohort I patients, unique non-cancer Read codes were counted if they occurred during the year before the cancer diagnosis date or the year after. For Cohort II patients, these codes were counted if they occurred within the two years before the patient’s death.

### Comparison of proportions of patients with prescriptions

To illustrate the pattern change of analgesic prescriptions for each of the analgesic groups (antineuropathics, any opioids, strong opioids and weak opioids), we calculated the percentage of patients being prescribed an item during the 12 months after cancer diagnosis for Cohort I patients across the year (by quarters) of diagnosis dates and the percentage of patients being prescribed an item during the 3 months before death for Cohort II patients across the year (by quarters) of death dates. Differences in the proportions of patients being prescribed an analgesic item for patients from different WIMD quintiles were analysed with *χ*^2^ test. Bonferroni multiple comparison corrections (×10) were applied to all *p*-values generated.

### Number of analgesic prescriptions prior to and during the pandemic

The numbers of analgesic prescriptions for each patient in the pre-pandemic period and during the pandemic were counted separately across the assessment periods (Fig. 1). Each patient’s total GP registration days were counted separately for the pre-pandemic period and the pandemic period and were used to adjust the number of prescriptions to N prescriptions per year (for Cohort I) and N prescriptions per 3 months (for Cohort II), respectively. These adjusted values represent the number of prescriptions per assessment period and were used for comparing the pre-pandemic and pandemic periods and for regression modelling analysis.

We designated 1^st^ April 2020 as the onset of the pandemic. April 2020 was the first full month during which social distancing measures were enacted and telemedicine was available in Wales, although these were both initiated in the latter half of March 2020 [11, 12].

### Statistical analysis for the impact of the pandemic on analgesic prescription

Multivariate logistic and Poisson regression modelling were applied to detect the impact of the pandemic on analgesic prescriptions after incorporating patients’ clinical details (cancer type and diagnosed stage (Cohort I only) [31], number of comorbidities (both Cohort I and Cohort II) and demographic factors, including age at diagnosis, sex, WIMD quintiles and rurality of residence at diagnosis for Cohort I and age at death, sex, WIMD quintiles and rurality of residence at death, and place of death for Cohort II. Logistic regression modelling was used to detect the impact of the pandemic on the likelihood of patients being issued a prescription during the assessment period. Poisson regression modelling was used to analyse the impact of the pandemic on the change of prescription quantities. Bonferroni multiple testing corrections (×4) were applied to all p-values generated. All analyses were carried out in R version 4.1.3 [32].

## Results

Characteristics of the two cohorts of patients are shown in Tables 1 and 2. There were 34,711 adult cases diagnosed with one of the four commonest cancers between Jan 2017 and Mar 2021 in Wales, of which, 25,252 cases (72.7%) survived at least 15 months and were included in Cohort I. Cohort II included 30,256 adult patients who died of any cancer in Wales between Jan 2018 and Mar 2022.

**Table 1:** Patient characteristics for Cohort I: patients diagnosed with a common cancer (female breast cancer, colorectal, non-small cell lung cancer (NSCLC) or prostate cancer) between Jan 2017 and Mar 2021 who survived the first 15 months post-diagnosis

	Cases diagnosed		Cases surviving the first 15 months		
	*N* diagnosed cases: 34,711	% of diagnosed cases	Cohort I cases: 25,252	% of Cohort I cases	% survival
*Year of diagnosis*					
2017	8236	23.7	6028	23.9	73.2
2018	8498	24.5	6221	24.6	73.2
2019	8521	24.5	6298	24.9	73.9
2020	7374	21.2	5198	20.6	70.5
2021 Q1	2082	6.0	1507	6.0	72.4
*Cancer type*					
Breast	8435	24.3	7846	31.1	93.0
Colorectal	8004	23.1	5530	21.9	69.1
Lung	8411	24.2	2874	11.4	34.2
Prostate	9861	28.4	9002	35.6	91.3
*Stage at diagnosis*					
Stage I	5832	16.8	5522	21.9	94.7
Stage II	7396	21.3	6831	27.1	92.4
Stage III	6120	17.6	4813	19.1	78.6
Stage IV	6017	17.3	2366	9.4	39.3
Unknown	9346	26.9	5720	22.7	61.2
*Age at diagnosis*					
18–49	2000	5.8	1788	7.1	89.4
50–59	4681	13.5	4032	16.0	86.1
60–79	20,731	59.7	15,610	61.8	75.3
80+	7299	21.0	3822	15.1	52.4
*Sex*					
Male	18,803	54.2	13,527	53.6	71.9
Female	15,908	45.8	11,725	46.4	73.7
*WIMD quintile*					
1 Most deprived	6376	18.4	4230	16.8	66.3
2	7019	20.2	4913	19.5	70.0
3	6742	19.4	4874	19.3	72.3
4	6995	20.2	5191	20.6	74.2
5 Least deprived	7579	21.8	6044	23.9	79.7
*Rurality*					
Urban	23,761	68.5	17,194	68.1	72.4
Rural	10,950	31.5	8058	31.9	73.6

**Table 2:** Patient characteristics for Cohort II: patients who died of cancer between Jan 2018 and Mar 2022

	*N* patients died: 30,256	% of patients died
*Year of death*		
2018	7148	23.6
2019	7171	23.7
2020	7192	23.8
2021	6948	23.0
2022 Q1	1797	5.9
*Place of death*		
Hospital	13,526	44.7
Hospice	2096	6.9
Home	11,997	39.7
Care home	2059	6.8
Other places	578	1.9
*Age at death*		
18–50	1094	3.6
51–60	2744	9.1
61–70	5910	19.5
71–80	10,206	33.7
81–90	8308	27.5
90+	1994	6.6
*Sex*		
Male	16,150	53.4
Female	14,106	46.6
*WIMD quintile*		
1 Most deprived	6174	20.4
2	6112	20.2
3	5958	19.7
4	5983	19.8
5 Least deprived	6029	19.9
*Rurality*		
Urban	20,832	68.9
Rural	9424	31.1

In Cohort I (Table 1), relatively less lung cancer patients (2,874, 11.4%) and less stage IV patients (2,366, 9.4%) were included due to the 15-month survivability criterion applied for this cohort. This cohort also contains less patients from the most deprived quintile (4230, 16.8%) and less patients from rural areas (8,058, 31.9%) than from urban areas (17,194, 68.1%).

Amongst the 30,256 patients included in Cohort II (Table 2), most died in hospital (13,526, 44.7%) or at home (11,997, 39.7%). Cohort II patients were evenly balanced across WIMD quintiles, and it contains a similar urban:rural ratio as Cohort I.

### Impact of the pandemic on the number of analgesic prescriptions and the likelihood of patients being prescribed an analgesic

**Fig. 2:**
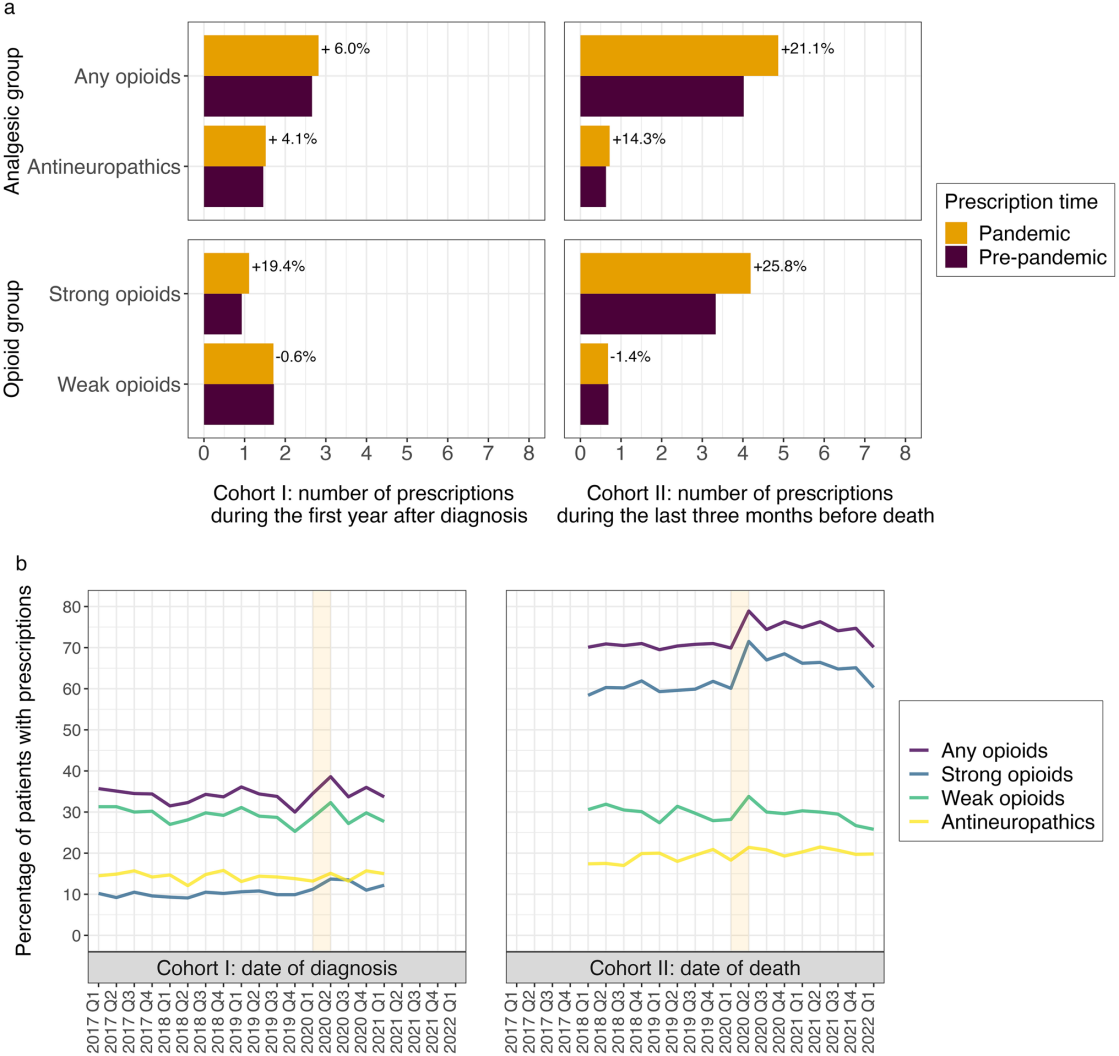
Average number of analgesic prescriptions during the pre-pandemic period and the pandemic period (**a**) and percentage of patients with analgesic prescriptions during the first year after the date of cancer diagnosis (Cohort I) and over the 3 months prior to the date of death (Cohort II) (**b**). The shaded strips in b mark the transition from the pre-pandemic to the pandemic in 2020

We observed that the pandemic led to a general increase in the number of analgesic prescriptions for both cohorts. As shown in Fig. 2a, amongst the two analgesic groups (any opioid and antineuropathics) and the two opioid sub-groups (strong and weak opioids), the greatest increases appeared in opioid analgesics for Cohort II patients, amongst whom the pandemic led to a 21.1% increase for any opioids (25.8% for strong opioids) in the average number of prescriptions during the last 3 months before death. Strong opioid prescriptions also increased for Cohort I patients during the year 1 post-diagnosis (19.4%, Cohort I in Fig. 2a). Small decreases were observed for weak opioids, with prescriptions dropping 0.6% and 1.4% respectively for Cohort I patients and Cohort II patients. The number of antineuropathic prescriptions during the pandemic increased for both cohorts, with a small increase of 4.1% for Cohort I patients and a larger increase of 14.3% for Cohort II patients (Fig. 2a).

Out of the 845 Cohort I patients diagnosed at the beginning of the pandemic (Q2 2020), 326 patients (38.6%) had an opioid prescription during their first year after diagnosis, including 116 patients (13.7%) with a prescription of strong opioids (Fig. 2b). These proportions are higher than the mean percentage of Cohort I patients diagnosed in the pre-pandemic period, which are 33.9% and 10.1% for any opioids and strong opioids respectively (Fig. 2b).

Higher proportions of Cohort II patients were prescribed an analgesic. 78.9% of Cohort II patients who died in Q2 2020 had an opioid prescription, an increase of 12.0% from the 70.5% of patients who died in the pre-pandemic period (Fig. 2b). The percentage of patients prescribed a strong opioid increased to 71.5% amongst patients who died in Q2 2020 compared with those who died in the pre-pandemic (60.2%), an 18.8% increase (Fig. 2b).

The proportions of patients with any opioid and strong opioid prescriptions return to pre-pandemic levels for Cohort I patients diagnosed at the end of Cohort I inclusion time (Q1 2021) and for Cohort II patients who died at the end of Cohort II inclusion time (Q1 2022) (Fig. 2b). For Cohort II patients, those whose death was during the pandemic (in or after Q2 2020) were more likely to be prescribed an antineuropathic agent compared to those whose death was pre-pandemic (pandemic 20.3% vs pre-pandemic 18.7%). This trend was not detected for Cohort I patients.

Logistic regression analysis was applied to detect the impact of the pandemic on the likelihood of patients being prescribed an analgesic. After patients’ clinical (cancer type and stage (Cohort I), number of non-cancer comorbidities) and demographic characteristics (age, sex, WIMD levels and rurality at diagnosis) were incorporated in the modelling, no statistically significant impact of the pandemic was detected on the likelihood of Cohort I patients being prescribed an opioid, strong opioid or antineuropathic item during year 1 post-diagnosis (Table 3). However, for Cohort II patients, the pandemic was associated with increased probability of opioid prescription (odds ratio (OR) = 1.17, 95% CI: 1.11–1.24, *p* < 0.001), strong opioids (OR = 1.22, 95% CI: 1.16–1.28, *p* < 0.001) and antineuropathics (OR = 1.09, 95% CI: 1.03–1.16, *p* = 0.009) (Table 3). The pandemic also significantly decreased the chance of weak opioid prescription for Cohort I patients (OR = 0.89, 95% CI: 0.84–0.94, *p* < 0.001), while for Cohort II patients, this effect was not significant.

**Table 3:** Impact of the pandemic on the likelihood of patients being prescribed an analgesic and on the number of analgesic prescriptions

		Impact of the pandemic on the likelihood of patients being prescribed an analgesic item*				Impact of the pandemic on the number of prescriptions*			
Patient cohort	Analgesics group	OR	95% CI	*p*	*p*_corrected_	IRR	95% CI	*p*	*p*_corrected_
Cohort I	Opioids	0.94	0.89, 0.99	0.026	0.104	1.03	1.02, 1.05	< 0.001	< 0.001
	Strong opioids	1.10	1.01, 1.20	0.035	0.142	1.15	1.12, 1.18	< 0.001	< 0.001
	Weak opioids	0.89	0.84, 0.94	< 0.001	< 0.001	0.97	0.95, 0.99	0.006	0.025
	Antineuropathics	0.98	0.92, 1.06	0.674	1.000	1.01	0.99, 1.04	0.220	0.879
Cohort II	Opioids	1.17	1.11, 1.24	< 0.001	< 0.001	1.04	1.03, 1.05	< 0.001	< 0.001
	Strong opioids	1.22	1.16, 1.28	< 0.001	< 0.001	1.06	1.04, 1.07	< 0.001	< 0.001
	Weak opioids	0.95	0.90, 1.00	0.035	0.139	0.95	0.93, 0.98	< 0.001	0.003
	Antineuropathics	1.09	1.03, 1.16	0.002	0.009	1.11	1.08, 1.14	< 0.001	< 0.001

*Analgesic prescriptions during the first year after the date of cancer diagnosis for Cohort I patients and analgesic prescriptions over the 3 months prior to the date of death for Cohort II patients

*OR*, odds ratio; *95% CI*, 95% confidence interval; *p*_corrected_, Bonferroni corrected *p*-value

We performed Poisson regression analysis by incorporating patients’ clinical and demographic characteristics to detect the impact of the pandemic on the quantity of analgesic prescriptions (Table 3). A similar association is seen in increased numbers of opioids and strong opioids prescribed for both cohorts with incidence rate ratio (IRR) = 1.03, 95% CI: 1.02–1.05, *p* < 0.001 for opioid prescription and IRR = 1.15, 95% CI: 1.12–1.18, *p* < 0.001 for strong opioid prescription for Cohort I patients and IRR = 1.04, 95% CI: 1.03–1.05, *p* < 0.001 for opioid prescription and IRR = 1.06, 95% CI: 1.04–1.07, *p* < 0.001 for strong opioid prescription for Cohort II patients. A highly significant increase in antineuropathic prescription during the pandemic was also found for Cohort II patients, with IRR = 1.11, 95% CI: 1.08–1.14, *p* < 0.001, while decreased prescriptions were found for weak opioids, with IRR = 0.97, 95% CI: 0.95–0.99, *p* = 0.025 for Cohort I patients and IRR = 0.95, 95% CI: 0.93–0.98, *p* = 0.003 for Cohort II patients (Table 3).

#### Prescription discrepancies amongst patients from different socioeconomic backgrounds

**Fig. 3:**
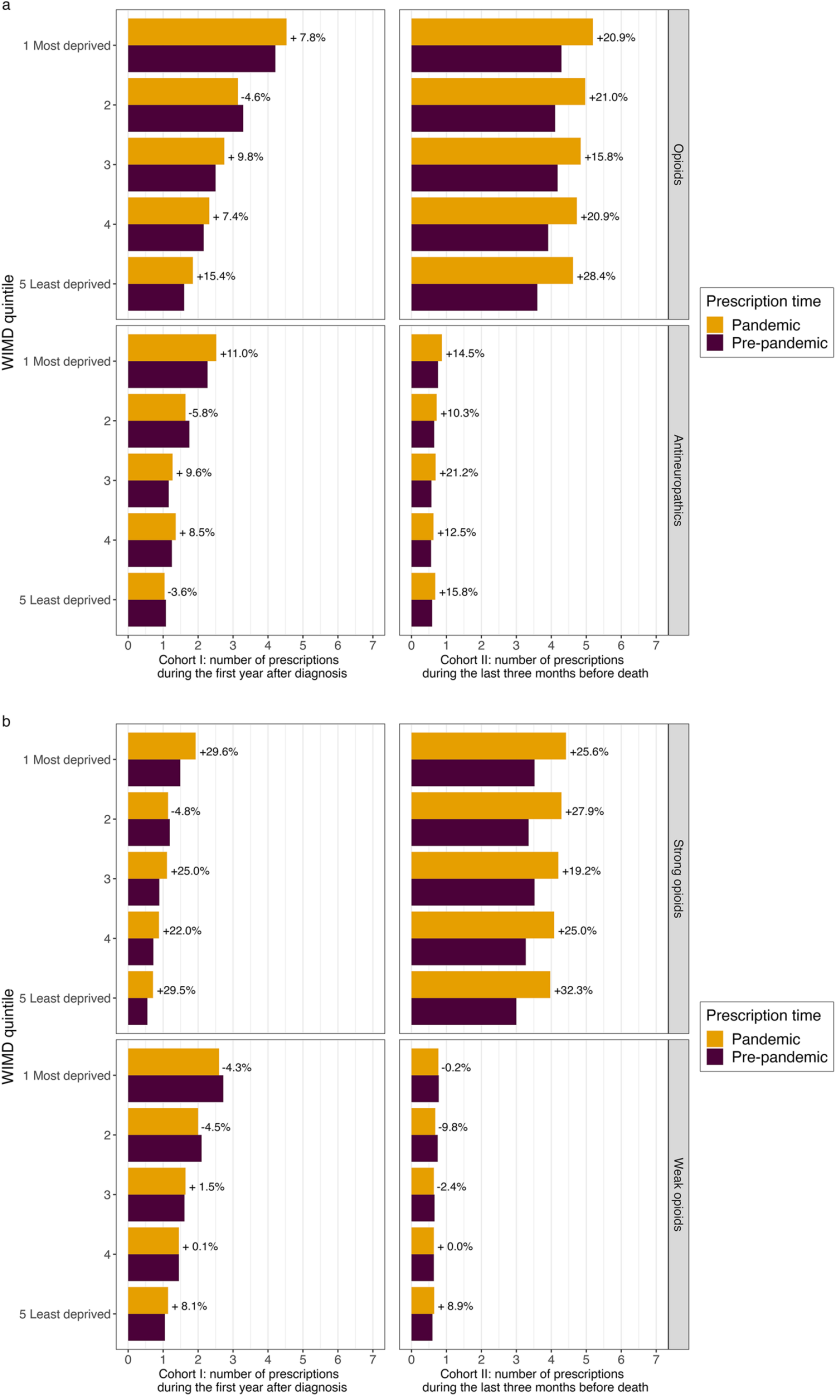
WIMD quintile of the residence areas and average analgesic prescriptions. **a** Average number of opioid and antineuropathic prescriptions. **b** Average number of strong and weak opioid prescriptions. Assessment time for Cohort I patients: the first year after the date of diagnosis; assessment time for Cohort II patients: the last 3 months before date of death

There was a tendency for Cohort 1 patients in the most deprived communities to have the highest number of analgesic prescriptions (Fig. 3)a during the first year post-diagnosis, and patients in the least deprived communities having the smallest number. This effect continued from the pre-pandemic to the pandemic period, and a general pattern of increased prescriptions during the pandemic period is observed (Fig. 3)a. These increases were higher for strong opioid prescriptions, with an increase of 29.6% for patients from the most deprived communities and an increase of 29.5% for patients from the least deprived communities (Cohort I in Fig. 3)b.

We observed consistent increases in analgesic prescription across different WIMD quintiles in the pandemic period for Cohort II patients, opioids (Fig. 3)a, strong opioids in particular (Fig. 3)b, and antineuropathics (Fig. 3)a.

Despite overall increases in strong opioid prescription for almost all the WIMD quintile levels during the pandemic period, weak opioid prescription did not show a consistent pattern of changes compared with the pre-pandemic period (Fig. 3)b.

**Fig. 4:**
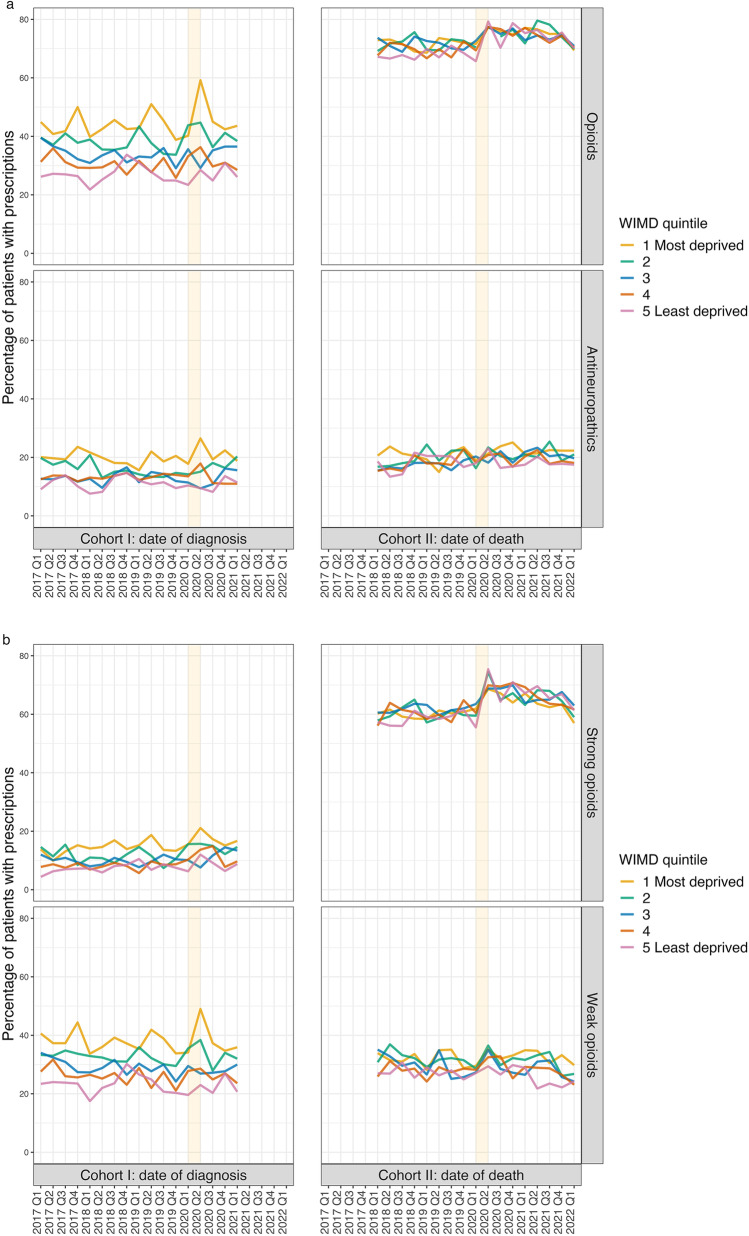
WIMD quintile of the residence areas and proportion of patients with an analgesics prescription. **a** Percentage of patients with opioids and antineuropathics prescription. **b** Percentage of patients with strong and weak opioids prescription. Assessment time for Cohort I patients: the first year after the date of diagnosis; assessment time for Cohort II patients: the last 3 months before the date of death. The shaded strips mark the transition from the pre-pandemic to the pandemic in 2020

We found that for Cohort I patients, the most deprived communities tended to consistently have the highest proportions of patients prescribed an analgesic item, while the least deprived communities generally had the lowest proportions (Cohort I in Fig. 4a—OR (level 1:Level 5) = 1.93, 95% CI: 1.78–2.09, *p* < 0.001 for opioids prescription; OR (level 1:level 5) = 1.80, 95% CI: 1.62–2.00, *p* < 0.001 for antineuropathics prescription).

The onset of the pandemic produced a notable increase in analgesic prescriptions for Cohort I patients from the most deprived communities (Fig. 4a and b), particularly for opioids with 59.2% of patients who were diagnosed during the first period of the pandemic (Q2 2020) being prescribed an item, representing a 47.3% uplift from the 40.2% for those diagnosed in the previous quarter (Q1 2020).

Socioeconomic variations in opioid prescription patterns were far less pronounced for Cohort II (Cohort II in Fig. 4a—OR (level 1:level 5) = 1.13, 95% CI: 1.04–1.23, *p* = 0.018 for opioids prescription; OR (level 1:level 5) = 1.10, 95% CI: 1.01–1.20, *p* = 0.144 for antineuropathics prescription), where despite the prescription levels across communities amongst those who died during Q2 2020 being far higher, the range was far more compressed compared to Cohort I (Fig. 4a and b).

## Discussion

In this national cohort study, we chose two distinct cohorts to evaluate the impact of the pandemic in different cancer care situations. Cohort II represents cancer patients in the last 3 months of life, who are likely to have specific oncological needs. Cohort I includes patients who survived 15 or more months from diagnosis and will include asymptomatic healthy patients, patients who have been radically treated, patients with chronic malignant disease of variable morbidity, and patients with actively relapsing disease which is either locally advanced or metastatic. In Cohort I, analgesia will be used for a variety of reasons, including non-cancer comorbidity. As an overall sample, Cohort I is a reasonable comparator for the more dynamic and focussed requirements of Cohort II patients.

We found that the COVID-19 pandemic was associated with a general increase in community prescription of opioid and antineuropathic analgesics for patients in Wales within the first 12 months of cancer diagnosis (Cohort I) and for cancer patients within 3 months of cancer-related death (Cohort II). Amongst the two broad analgesic groups, and the two opioid sub-groups (strong and weak opioids) evaluated, significant increases in the quantity of prescriptions were identified in both opioid (strong opioids especially) and antineuropathic prescriptions for both cohorts. A significantly higher proportion of patients within 3 months of cancer-related death were given strong opioids and antineuropathics during the pandemic period, when compared to pre-pandemic. Prescription of weak opioids showed a decreasing trend in the pandemic period. These findings are consistent with changes in community prescribing to compensate for pandemic-related service changes [14, 33], including reduced access to specialist hospital-based cancer services and/or reduced specialist supervision and medication chart rationalisation, and an emphasis on primary care-based palliation. Further investigation of secondary care analgesic use will be beneficial in interpreting these results more fully.

We observed a spike in opioid prescription for Cohort I patients diagnosed in Q2 2020 and for Cohort II patients who died in Q2 2020. This appears to be an isolated effect, coinciding with the start of UK pandemic measures. Similar spikes in non-cancer patients have been recorded in Wales for non-analgesic community prescriptions [34], and in England for both analgesic and non-analgesic community prescriptions [35], and the hypothesis is that this represents a form of stockpiling in anticipation of reduced availability. These spikes may be the result of either patient-initiated prescription requests, a more systematic primary care initiative, or a combination of the two. The prescription spikes do not necessarily equate to an increase in consumption.

Our study is able to monitor sequential repeated prescriptions over time in primary care. It is perceived that each sequential record for prescription occurs when patients are running out of drugs, which is a proxy for daily usage and adherence of drug items prescribed for managing cancer-related pains.

Prescription patterns are influenced by socioeconomic conditions [35, 36], and our data are consistent with this. For Cohort II patients, socioeconomic variations were less pronounced and the range was more compressed compared to Cohort I. We hypothesise that this is due to closer supervision of patients with increasingly complex and dynamic needs and demands towards the end of life. In this setting, there will likely be a regular review of medication charts, and analgesic prescription will likely be consistently applied and mainly tailored towards cancer-specific symptoms. In contrast, the variation seen in Cohort I likely reflects a combination of looser medication supervision, and a wider range of symptomatology, including analgesia for pain not directly attributable to cancer, or caused by non-malignant comorbidity, which also displays a social gradient.

### Study limitations

Paracetamol (acetaminophen) and non-steroidal anti-inflammatory drugs are not included in this analysis, despite being some of the most commonly used analgesics. These may be prescribed, but are commonly bought across the counter, so usage is only partially ascertained in prescription analysis. Tablets or capsules containing codeine 8mg plus paracetamol 500mg may also be purchased without a prescription in the UK. In addition, this study only looks at the number of discrete analgesic prescriptions.

While Defined Daily Doses (DDDs) are a more robust measure for prescribed items and the presence of it in the electronic records adds to the granularity of the data included in the current analysis, our focus in this study was to monitor the impact of the pandemic on prescription of each drug category. Further research on adherence and individual base item usage per day would add to the insights provided in this study.

Some of these patients will have received hospital or hospice prescriptions, which are not captured in this study.

## Conclusion

With national-scale linked data, we demonstrate significant changes to community analgesic prescription patterns for cancer patients over the course of the UK pandemic, superimposed upon pre-existing sociodemographic variation, with increased opioid and antineuropathic prescriptions for both newly diagnosed patients and patients receiving end-of-life care. The latter patient population also had a higher chance of receiving an analgesic prescription compared to pre-pandemic times. These effects reflect UK and Wales pandemic-related healthcare policy changes, as well as local primary care coping strategies, and changes in health-seeking behaviour.

## Supplementary information

ESM 1 (DOCX 36 kb)

## Data Availability

The main individual-level data sources used in this study are available in the SAIL Databank at Swansea University, Swansea, UK, but as restrictions apply they are not publicly available. All proposals to use SAIL data are subject to review by an independent Information Governance Review Panel (IGRP) which includes members of the public and external experts in data security. Before any data can be accessed, approval must be given by the IGRP. The IGRP gives careful consideration to each project to ensure the proper and appropriate use of SAIL data. When access has been granted, it is gained through a privacy-protecting safe haven and remote access system referred to as the SAIL Gateway. SAIL has established an application process to be followed by accredited bona fide researchers to access data for approved research purposes at https://www.saildatabank.com/application-process/.
