# Supplementary material for: The impact of the COVID-19 pandemic on community prescription of opioid and antineuropathic analgesics for cancer patients in Wales, UK
Source: Support Care Cancer. 2023 Aug 22;31(9):531. doi: 10.1007/s00520-023-07944-8 (PMC10444652; doi:10.1007/s00520-023-07944-8)
Supplement: Supplementary file 1 — (DOCX 36 kb) [file 520_2023_7944_MOESM1_ESM.docx]

**The Impact of the COVID-19 pandemic on community prescription of opioid and antineuropathic analgesics for cancer patients in Wales, UK**

**Supplementary Information**

**Supplementary Table 1 Analgesic groups and names of analgesics included in the study [1-5].**

| **Analgesic Group** | **Analgesic Subgroup** | **Medicine Name** |
| --- | --- | --- |
| Opioids | Strong opioids | *Alfentanil* |
|  |  | *Buprenorphine* |
|  |  | *Diamorphine* |
|  |  | *Fentanyl* |
|  |  | *Hydromorphone* |
|  |  | *Methadone* |
|  |  | *Morphine* |
|  |  | *Oxycodone* |
|  |  | *Pethidine* |
|  |  | *Tapentadol* |
|  | Weak opioids | *Codeine* |
|  |  | *Dihydrocodeine* |
|  |  | *Tramadol* |
|  |  | *Meptazinol* |
|  |  | *Compounds of aspirin and weak opioid* |
|  |  | *Compounds of paracetamol and weak opioid* |
| Antineuropathics | Antineuropathics | *Pregabalin* |
|  |  | *Gabapentin* |
|  |  | *Amitriptyline* |
|  |  | *Duloxetine* |

**References:**

1. National Institute for Health and Care Excellence (2022) British National Formulary (BNF). URL: <https://bnf.nice.org.uk/>. Accessed 15 Aug 2023
2. World Health Organization (1986) Cancer pain relief. URL: <https://apps.who.int/iris/handle/10665/43944>. Accessed 28 Feb 2022
3. World Health Organization (2019) WHO guidelines for the pharmacological and radiotherapeutic management of cancer pain in adults and adolescents. URL: https://www.who.int/publications/i/item/97892415503908. Accessed 14 Feb 2022
4. Fallon M, Giusti R, Aielli F *et al* (2018) Management of cancer pain in adult patients: ESMO Clinical Practice Guidelines. *Annals of Oncology* 29:iv166–iv191. Accessed 14 Feb 2022
5. National Institute for Health and Care Excellence (2021) Clinical Knowledge Summaries - Analgesia - mild-to-moderate pain. URL: https://cks.nice.org.uk/topics/analgesia-mild-to-moderate-pain/management/weak-opioids/. Accessed 14 Feb 2022
